# Preliminary opinion on assessment categories of stomach ultrasound report and data system (Su-RADS)

**DOI:** 10.1007/s10120-018-0798-x

**Published:** 2018-01-25

**Authors:** Zhining Liu, Weidong Ren, Jintao Guo, Ying Zhao, Siyu Sun, Yuhong Li, Zhijun Liu

**Affiliations:** 1 Ultrasound Department, First Affiliated Hospital of JinZhou Medical University, Jinzhou, Liaoning People’s Republic of China; 2 Ultrasound Department, Shengjing Hospital of China Medical University, Shenyang, Liaoning People’s Republic of China; 3 Endoscopy Center, Shengjing Hospital of China Medical University, Shenyang, Liaoning People’s Republic of China; 4 General Surgical Department, Shengjing Hospital of China Medical University, Shenyang, Liaoning People’s Republic of China

**Keywords:** Gastric cancer, Mass screening, Transabdominal ultrasound, Oral contrast agent, Stomach ultrasound report and data system

## Abstract

**Objective:**

Transabdominal ultrasound after oral administration of an echoic cellulose-based gastric ultrasound contrast agent (TUS-OCCA) has recently been suggested as a valuable mass-screening tool for gastric cancer. The aim of this study was to propose a producible stomach ultrasound reporting and data system (Su-RADS) using TUS-OCCA for gastric cancer screening.

**Patients:**

The study includes information of 2738 patients who underwent both gastroscopy and TUS-OCCA examinations recorded in software system. Gastroscopy examination with pathological diagnosis was considered as gold standard. Various gastric lesions were classified into category 1–5 based on gastric wall thicknesses of them (especially the mucosa layer).

**Results:**

The total malignant ratios of patients enrolled in this study were 17.1% (469/2738). The malignant ratios for category 1–5 were, respectively, 1.1, 1.7, 12.2, 34.2 and 78.1%. Category 2 indicated mild thickening of gastric wall at low risk for malignancy (1.7%); category 3 indicated moderate thickening at moderate risk for malignancy (12.2%); category 4 indicated severe thickening at high risk for malignancy (34.2%); category 5 indicated extremely severe thickening at extremely high risk for malignancy (78.1%). If category 2 was identified as cut-off point distinguishing between benign and malignant, the sensitivity and specificity by Su-RADS are 95.1 and 78.6%, respectively.

**Conclusion:**

The Su-RADS system could inform the physicians about key findings, indicating the risk for malignancy and necessity of additional gastroscopy examination. Prospectively randomly controlled study design with larger clinical trial is needed for further investigations.

**Electronic supplementary material:**

The online version of this article (10.1007/s10120-018-0798-x) contains supplementary material, which is available to authorized users.

## Introduction

Gastric cancer remains the third leading cause of cancer mortality in the world [1]. The use of gastroscopy for opportunistic screening of gastric cancers is widely accepted, while the use of this procedure for mass screening of gastric cancers remains questionable, even in developed countries such as Japan [1–4]. In addition, the rates of missed lesion by endoscopic examination have been reported to be about 10–31% [1, 5–7]. Moreover, mass screening for gastric cancer by gastroscopy examination might increase the risk of cross-infection. Hence, there is a need for a technique for mass screening of gastric cancer that is relatively safe, simple, inexpensive, and reliable.

In the light of the remarkable advances in ultrasound technology, transabdominal ultrasound after oral administration of an echoic, cellulose-based, gastric ultrasound contrast agent (TUS-OCCA) has recently been suggested as a valuable mass-screening tool for gastric lesions in selected patients, where the incidence of gastric cancer is high and the body habitus of the population is more suitable [8–15]. Moreover, there is a lack of a producible diagnostic criteria and risk assessment system for gastric cancer screening by TUS-OCCA. Thus, in the present study, we propose the stomach ultrasound reporting and data system (Su-RADS) which could be objective and easily comprehend.

## Materials and methods

### Study patients

The study was approved by the ethics committee at Shengjing Hospital of China Medical University and informed consents were obtained from all patients. From May 1st, 2012 to May 1st, 2017, patients who had underwent gastroscopy examination in our hospital or our affiliated facilities within 2 weeks and agree to undergo TUS-OCCA examination without charge were enrolled. We use the NEUPACS software systems at our hospital, which collect data in pathological/endoscopy and ultrasound department. Gastroscopy examination with pathological diagnosis was considered as gold standard. For statistical analyses in this study, we exclude the patients with gastric lesions present as solitary masses (gastric polyps and gastric submucosal tumors).

### Cellulose-based oral contrast agent

The commercially available oral contrast agent (50 g per package) (Best; East Asia Medical Products Co, Ltd, Huzhou, Zhejiang, China) or (Dongbeide; Zhongdaoaode Medical Products Co, Ltd, Beijing, China) was reconstituted in 500 mL of boiling water to form a homogeneous thin paste. The paste was cooled to a suitable temperature and was then administered orally to facilitate distension of the stomach. This cellulose-based oral contrast agent was slightly sweet, with a pleasant taste that was generally acceptable to the patient. The acoustic velocity and specific acoustic impedance of the contrast agent were similar to those of liver tissue, and the contrast-filled stomach had a homogeneous appearance with a mid-high level echogenicity. No antispasmodics were used. Each patient was encouraged to drink the entire 500 mL of contrast solution; in the few cases where this was not tolerable to the patient, a smaller volume was acceptable.

### Scanning procedure

**Fig. 1:**
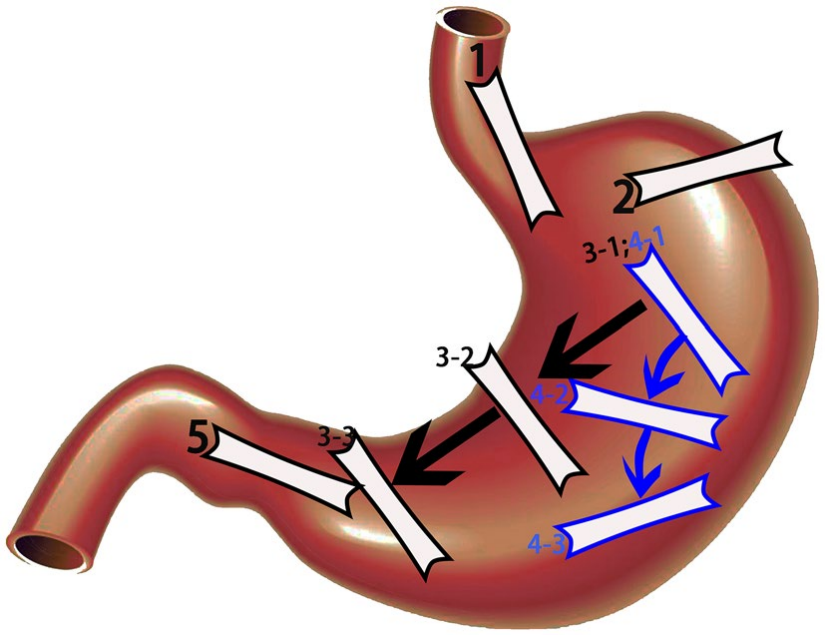
The entire stomach was scanned in five steps. Step 1, which was mainly for scanning the cardia. Step 2, which was mainly for scanning the gastric fundus. Step 3, for scanning the gastric fundus, body, and antrum in serial transverse section. Step 4, for scanning the fundus, body, and antrum in serial coronal section. Step 5, which was for scanning the antrum and pylorus. Steps 3 and 4 were the key steps, respectively, obtaining serial transverse and coronal sections of the whole stomach, including the gastric fundus, body, angle, and antrum

The entire stomach was scanned in 5 steps (Fig. 1). Step 1, which was mainly for scanning the cardia, was performed by moving the probe from the xiphoid process to the left costal arch with the patient in a supine position. Step 2, which was mainly for scanning the gastric fundus, was performed by placing the probe at the left 10th intercostal space. Step 3, for scanning the gastric fundus, body, and antrum in transverse section, was performed by moving the probe from the left costal arch along the outline of the stomach with the patient in the right decubitus position. Step 4, for scanning the fundus, body, and antrum in coronal section, was performed by rotating the probe along the left costal arch using the caudal end of the probe as an axis, and simultaneously tilting the probe about 45°, with the patient in the right decubitus position. Step 5, which was for scanning the antrum and pylorus, was performed by placing the probe vertical to the right costal arch with the patient in the supine position. Steps 3 and 4 were the key steps, respectively, obtaining serial transverse and coronal sections of the whole stomach, including the gastric fundus, body, angle, and antrum.

**Fig. 2:**
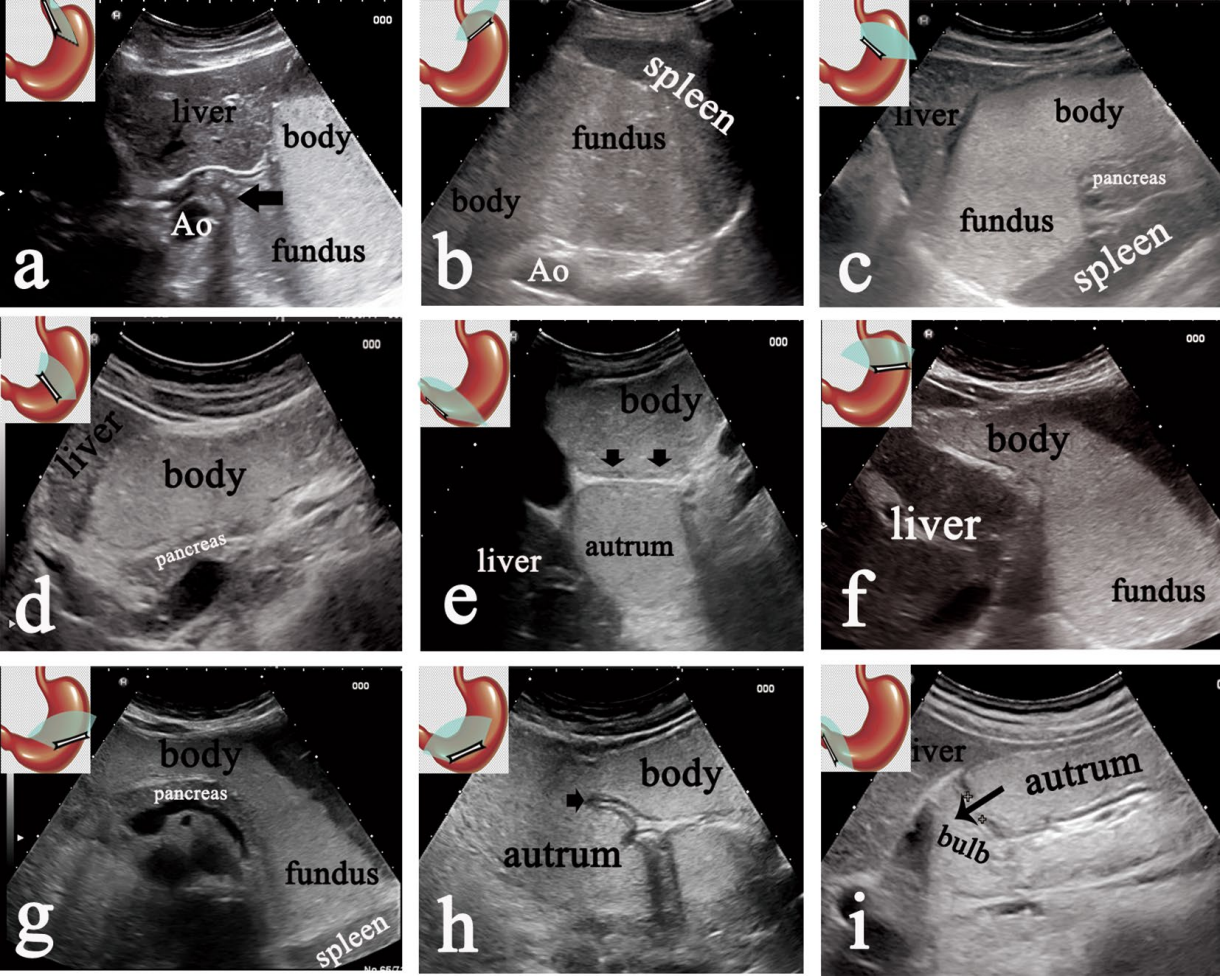
Category 1: normal finding. Normal finding in nine standard sections obtained by the 5 steps. **a** Normal sagittal sections of the gastric cardia (arrow) and fundus obtained at Step 1. **b** Normal short section of the gastric fundus by Step 2. **c**–**e** Normal serial transverse sections of gastric fundus, body, angle (arrow) and antrum by Step 3-1, 3-2 and 3-3, respectively. **f**–**h** Normal serial coronal section of the gastric fundus, body, angle (arrow) and antrum by Step 4-1, 4-2 and 4-3, respectively. **i** Normal longitudinal section of the gastric antrum and pylorus by Step 5

**Fig. 3:**
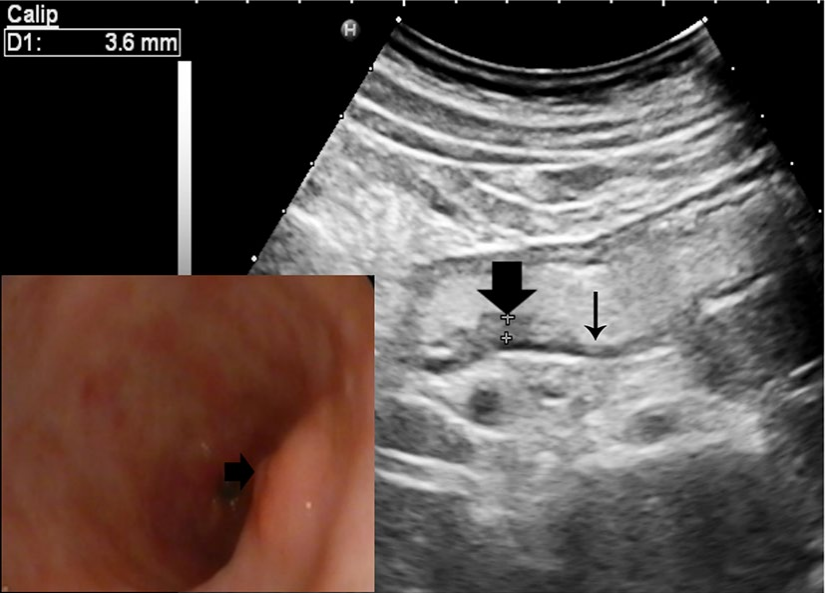
Category 4A: suspicious malignant finding. TUS-OCCA examination showing gastric mucosa thickness was 3.6 mm (thick arrow) and the surrounding gastric wall was thinning about 3 mm (thin arrow). Gastroscopy examination showing the gastric mucosa was red and white with local eminence (arrow), pathological diagnosis revealed atrophic gastritis with high-grade intraepithelial neoplasia

Nine pictures (referring to Fig. 2) of the stomach should be obtained by five steps. As has been established, the majority of gastric cancers involve the lesser curvature (including pylorus, angle, and cardia) and the gastric antrum. Hence, the thickness of the gastric wall of the whole stomach should be measured at five points as following: (1) lower segment of the esophagus adhere to the cardia (Fig. 2a); (2) gastric fundus adhere to the cardia (Fig. 2b); (3) less curvature of the gastric body adhere to the cardia (Fig. 2f); (4) gastric angle (Fig. 2h); (5) gastric antrum adhere to the pylorus (Fig. 2i). In addition, if the local thickness of gastric wall was thicker than the surrounding gastric wall, this point should also be measured.

The thicknesses of gastric wall of normal stomach, acute and chronic gastritis, gastric ulcer, gastric high-grade intraepithelial neoplasia, gastric cancer, and gastric lymphoma were measured, respectively.

## Results

### Study patients

The study includes information of 2738 patients who underwent both gastroscopy and TUS-OCCA examinations recorded in NEUPACS system. The detection rate of TUS-OCCA for chronic gastritis was 81.1% (643/793); the detection rate for acute hemorrhagic erosive gastritis was 100% (12/12); the detection rate for benign gastric ulcer was 79% (259/328); the detection rate for gastric high-grade intraepithelial neoplasm and early gastric cancer was 57.4% (78/136); the detection rate for advanced gastric cancer was 97.5% (316/324); the detection rate for gastric lymphoma was 100% (9/9).

As shown in Table 1, for normal stomach (1136 cases), the mucosal layer thicknesses on TUS-OCCA assessment were about 1.4 mm (median, range 1–1.6 mm); the full thickness of gastric wall in the antrum was 4.9 mm (median, range 4.3–6.2 mm), in the body was 4.0 mm (median, range 3.3–4.4 mm), in the fundus was 3.0 mm (median, range 2.1–3.5 mm). For chronic gastritis (643 cases), the mucosal layer thicknesses were 1.9 mm (median, range 1.5–4.3 mm); the full thickness of gastric wall was 6.3 mm (median, range 5.7–9.3 mm). For acute gastritis (12 cases), the mucosal layer thicknesses were 5.4 mm (median, range 3.3–7.4 mm); the full thickness of gastric wall was 10.6 mm (median, range 8.2–12.8 mm). For benign gastric ulcer (259 cases), the mucosal layer thicknesses were 4.1 mm (median, range 2.6–8.2 mm); the full thickness of gastric wall was 9.3 mm (median, range 6.4–16.3 mm). For gastric high-grade intraepithelial neoplasia and early gastric cancer (78 cases), the mucosal layer thicknesses were 3.4 mm (median, range 2.4–5.8 mm); the full thickness of gastric wall was 9.1 mm (median, range 6.8–11.3 mm). For advanced gastric cancer (316 cases), the full thickness of gastric wall was 16.8 mm (median, range 9.2–34.8 mm). For gastric lymphoma (9 cases), the full thickness of gastric wall was 18.1 mm (median, range 8.2–38.9 mm).

**Table 1:** Data collected from NEUPACS software systems in pathological, endoscopy and ultrasound department

Final diagnosis (subjects, *n*)	Detection rates by TUS-OCCA	Mucosal thicknesses median (range, mm)/*n*	Full thickness median (range, mm)/*n*	Su-RADS categories					
				0	1	2	3	4	5
Normal (1136)	–	1.4(1–1.6)/1136	4.9(4.3–6.2)/1136 for antrum 4.0(3.3–4.4)/1136 for body 3.0(2.1–3.5)/1136 for fundus	132	841	152	11	0	0
Chronic gastritis (793)	643/793(81.1%)	1.9(1.5–4.3)/643	6.3(5.7–9.3)/643	82	68	506	124	13	0
Acute hemorrhagic erosive gastritis (12)	12/12(100%)	5.4(3.3–7.4)/12	10.6(8.2–12.8)/12	0	0	0	0	3	9
Benign gastric ulcer (328)	259/328(79%)	4.1(2.6–8.2)/259	9.3(6.4–16.3)/259	21	12	20	16	184	75
GHIN and early gastric cancer (136)	78/136(57.4%)	3.4(2.4–5.8)/78	9.1(6.8–11.3)/78	18	10	11	19	65	13
Gastric advanced cancer (324)	316/324(97.5%)	–	16.8(9.2–34.8)/316	5	0	1	2	37	279
Gastric lymphoma (9)	9/9(100%)	–	18.1(8.2–38.9)/9	0	0	0	0	2	7
Total									
Total (2738)				258	931	690	172	304	383

Various gastric lesions were classified into category 1–5 based on gastric wall thicknesses of them (especially the mucosa layer). Category 1–5 were proposed as following: category 1 indicate almost normal finding; category 2 indicate mild thickening of gastric wall at low risk for malignancy; category 3 indicate moderate thickening at moderate risk for malignancy; category 4 indicate severe thickening at high risk for malignancy; category 5 indicate extremely severe thickening at extremely high risk for malignancy. The total malignant ratios of patients enrolled in this study were 17.1% (469/2738), referring to Table 2.

**Table 2:** Su-RADS categories distinguishing between benign and malignant

Su-RADS categories	TUS-OCCA examinations (*n*)	Final diagnosis		Malignant ratio (%)
		Benign	Malignant	
0	258	235	23	8.9
1	931	921	10	1.1
2	690	678	12	1.7
3	172	151	21	12.2
4	304	200	104	34.2
5	383	84	299	78.1
Total	2738	2269	469	17.1

## Su-RADS assessment categories

### Category 1: Almost normal finding

This is almost a normal finding on TUS-OCCA: gastric mucosa thickness is less than 1.5 mm. If the layering of gastric wall could not be demonstrated clearly, we measure the full thickness of the gastric wall. Assessment category 1 may also be used in a diagnostic TUS-OCCA report, when the full thickness of the gastric antrum and cardia is less than 5 mm, gastric body is less than 4 mm and gastric fundus is less than 3 mm.

For the patients enrolled in this study, the malignant ratio for category 1 was 1.1% (10/931); few of GHIN (gastric high-grade intra epithelial neoplasm) and early gastric cancer were unavoidably classified into category 1.

### Category 2: Mild thickening of gastric wall at low risk for malignancy

Category 2 assessments indicate that there is mild thickening of gastric wall: gastric mucosa thickness is about 1.5–2 mm. If the layering of gastric wall could not be demonstrated clearly, we measure the full thickness of the gastric wall. Assessment category 2 may also be used in a diagnostic TUS-OCCA report, when the full thickness of the gastric antrum and cardia is 5–6 mm, gastric body is 4–5 mm and gastric fundus is 3–4 mm.

For the patients enrolled in this study, the malignant ratio for category 2 was 1.7% (12/690). This is almost a gastritis (mild degree) finding on TUS-OCCA. Few of GHIN (gastric high-grade intra epithelial neoplasm) and early gastric cancer were unavoidably classified into category 2.

### Category 3: Moderate thickening at moderate risk for malignancy

Category 3 assessments indicate that there is moderate thickening of gastric wall: gastric mucosa thickness is about 2–2.5 mm. If the layering of gastric wall could not be demonstrated clearly, we measure the full thickness of the gastric wall. Assessment category 3 may also be used in a diagnostic TUS-OCCA report, when the full thickness of the gastric antrum and cardia is 6–7 mm, gastric body is 5–6 mm and gastric fundus is 4–5 mm.

A total of 172 patients were classified into category 3, the malignant ratio for category 3 was 12.2% (21/172) including normal (11), chronic gastritis (124), benign gastric ulcer (16), gastric high-grade intraepithelial neoplasia and early gastric cancer (19), advanced gastric cancer (2).

### Category 4: Severe thickening at high risk for malignancy

This is a suspiciously malignant finding on TUS-OCCA: gastric mucosa thickness is about 2.5–5 mm. If the layering of gastric wall could not be demonstrated clearly, we measure the full thickness of the gastric wall. Assessment category 4 may also be used in a diagnostic TUS-OCCA report, when the full thickness of the gastric antrum and cardia is 7–10 mm, gastric body is 6–9 mm and gastric fundus is 5–8 mm. In addition, if the thickening of gastric wall does not reach these criteria above, category 4 may also be used if the continuity of gastric submucosal layer was interrupted by hypoechoic area. Category 4 can be subdivided into 4A and 4B, according to the presence or absence of mucosa ulceration in the surface of thickening gastric wall. The absence of mucosa ulceration could be subdivided into 4A (referring to Fig. 3, Electronic Supplementary Material-Figure S1); the presence of mucosa ulceration could be subdivided into 4B (Electronic Supplementary Material-Figure S2).

A total of 304 patients were classified into category 4, the malignant ratio for category 4 was 34.2% (104/304) including normal (0), chronic gastritis (13), acute gastritis (3), benign gastric ulcer (184), gastric high-grade intraepithelial neoplasia and early gastric cancer (65), advanced gastric cancer (37) and lymphoma (2).

### Category 5: Extremely severe thickening at extremely high risk for malignancy

This is a highly suggestive of malignant finding on TUS-OCCA: gastric mucosa thickness is more than 5 mm. If the layering of gastric wall could not be demonstrated clearly, we measure the full thickness of the gastric wall. Assessment category 5 may also be used in a diagnostic TUS-OCCA report, when the full thickness of the gastric antrum and cardia is more than 10 mm, gastric body is more than 9 mm and gastric fundus is more than 8 mm. Category 5 can be subdivided into 5A and 5B, according to the presence or absence of mucosa ulceration in the surface of thickening gastric wall. The absence of mucosa ulceration could be subdivided into 5A; the presence of mucosa ulceration could be subdivided into 5B (referring to Electronic Supplementary Material-Figure S3).

A total of 383 patients were classified into category 5, the malignant ratio for category 5 was 78.1% (299/383) including normal (0), chronic gastritis (0), acute hemorrhagic erosive gastritis (9), benign gastric ulcer (75), gastric high-grade intraepithelial neoplasia and early gastric cancer (13), advanced gastric cancer (279) and lymphoma (7).

## Su-RADS assessment categories indicating the risk for malignancy and the necessity of additional gastroscopy examination (referring to Table 3)

### Assessment is incomplete

#### Category 0: Incomplete—need additional gastroscopy examination

There is a finding for which additional examinations (including endoscopy) are needed, when TUS-OCCA screening examination could not identify weather there is a lesion in the stomach. Under certain circumstances, assessment category 0 may be used in a diagnostic TUS-OCCA report, such as when: (1) the ultrasound image quality is poor due to unsuitable body habitus, e.g., obesity, high diaphragmatic position, Gastric stump, interference by colon gas, and etcetera; (2) the stomach cavity is poor filling if the patient did not orally administrated enough oral contrast agent. In our experience, TUS-OCCA examination is not satisfactory in 10% of the population of in Northeast China. Hence, the disadvantages of TUS-OCCA screening should be informed. For the population whose body habitus is not suitable for TUS-OCCA screening, gastroscopy examination or other screening tool are needed.

**Table 3:** Su-RADS assessment categories and the necessity of additional gastroscopy examination

Assessment	TUS-OCCA report	Necessity of additional gastroscopy
Category 0: Incomplete–need Additional examination	Not satisfactory	Necessary
Category 1: Almost normal finding	No gastric wall thickening	May be unnecessary
Category 2: Low risk for malignancy	Mild thickening of gastric wall	Might be unnecessary
Category 3: Moderate risk for malignancy	Moderate thickening of gastric wall	May be necessary
Category 4: High risk for malignancy	Severe thickening of gastric wall with or without ulceration	Necessary and biopsy should be performed
Category 5: Extremely high risk for malignancy	Extremely severe thickening of gastric wall with or without ulceration	Necessary and multiple endoscopy biopsies must be performed
Category 6: Known biopsy-proven malignancy	Corresponded with gastric cancer	Surgical excision or other treatment

This assessment categories were used for gastric lesions presenting as gastric wall thickened, gastric lesions present as solitary masses were not included (gastric polyps and gastric submucosal tumors)

### Assessment is complete—final categories

#### Category 1: Almost normal finding—additional gastroscopy examination may be unnecessary

Category 1 assessments indicate that there is no gastric wall thickening. Note that although category 1 indicates that additional gastroscopy examination may be unnecessary, it should also be followed by the management recommendation for routine health check-up program. Moreover, if the patient has symptoms and signs in the screening interval, additional examinations (including endoscopy) are still needed. However, some gastric cancer in the early stage may not present as thickening of gastric wall.

#### Category 2: Low risk for malignancy—additional gastroscopy examination might be unnecessary

Note that although category 2 indicates that additional gastroscopy examination might be unnecessary, it should be followed by the management recommendation for routine health check-up program. If the patient has symptoms and signs in the screening interval, additional examinations (including endoscopy) are still needed. However, some gastric cancer in the early stage may not present as thickening of gastric wall.

#### Category 3: Moderate risk for malignancy—additional gastroscopy examination may be necessary

For category 3 assessments, additional gastroscopy examination may be necessary; for the patients did not willing to undergo gastroscopy examination, short-interval (3-month) follow-up should be undergone. If the patient has symptoms and signs in the short-interval, additional examinations (including endoscopy) are needed.

#### Category 4: High risk for malignancy—additional gastroscopy examination is necessary and biopsy should be performed

For category 4 assessments, gastroscopy examination is necessary and biopsy should be performed.

#### Category 5: Extremely high risk for malignancy—additional gastroscopy examination is necessary and multiple biopsies must be performed

For category 5 assessments, gastroscopy examinations are necessary and multiple endoscopy biopsies must be performed.

#### Category 6: Known biopsy-proven malignancy

The category 6 is reserved for examinations performed after biopsy proof of malignancy (imaging performed after percutaneous biopsy but prior to surgical excision). TUS-OCCA is mainly used for the evaluation of gastric cancer staging and lymph node metastasis.

## Sensitivity and specificity by Su-RADS categories distinguishing between benign and malignant

As shown in Table 4, if category 1 was identified as cut-off point, the sensitivity and specificity by Su-RADS categories were 97.8 and 45.3%, respectively; if category 2 was identified as cut-off point, the sensitivity and specificity by Su-RADS categories were 95.1 and 78.6%, respectively; if category 3 was identified as cut-off point, the sensitivity and specificity by Su-RADS categories were 90.4 and 86%, respectively; if category 4 was identified as cut-off point, the sensitivity and specificity by Su-RADS categories were 67 and 95.9%, respectively.

**Table 4:** Sensitivity and specificity by Su-RADS categories distinguishing between benign and malignant

Su-RADS	Categories	Final diagnosis		Sensitivity (%)	Specificity (%)
		Benign (−)	Malignant (+)		
Category 1 as cut-off point					
Category 1	931	921(TN)	10(FN)	97.8	45.3
Category 2–category 5	1549	1113(FP)	436(TP)		
Total	2480	2034	446		
Category 2 as cut-off point					
Category 1–category 2	1621	1599(TN)	22(FN)	95.1	78.6
Category 3–category 5	859	435(FP)	424(TP)		
Total	2480	2034	446		
Category 3 as cut-off point					
Category 1–category 3	1793	1750(TN)	43(FN)	90.4	86
Category 4–category 5	687	284(FP)	403(TP)		
Total	2480	2034	446		
Category 4 as cut-off point					
Category 1–category 4	2097	1950(TN)	147(FN)	67	95.9
Category 5	383	84(FP)	299(TP)		
Total	2480	2034	446		

*TP* true positive, *FP* false positive, *TN* true negative, *FN* false negative

## Discussion

Although various imaging modalities can be used to detect gastric lesions, including endoscopy, barium studies, computed tomography, magnetic resonance imaging, and ultrasound, no suitable mass-screening tool for gastric cancer has been recommended by the World Health Organization.

The use of gastroscopy for opportunistic screening of gastric cancers is widely accepted, while the use of this procedure for mass screening of gastric cancers is not recommended by the World Health Organization. Since population-based mass-screening technique should be safe, simple, inexpensive and reliable, gastroscopy examination does not appear to be a perfect mass-screening tool for gastric cancer [4]. Gastroscopy examination is an invasive approach and not easily tolerated by the subjects; it also carry the risk of cross-infection; the amount of endoscopic equipment and endoscopist cannot easily meet the needs of mass-screening. Moreover, the rates of missed lesion by endoscopic examination have been reported to be about 10–31% [1, 5–7], especially for the gastric cancers without distinct mucosa changes (e.g., Linitis plastica). Choi et al. [7] analyzed data on 924,822 men and women in Korea who underwent endoscopy mass screening for gastric cancer. The sensitivity of endoscopy screening to detect gastric cancer was only 69% (2415 gastric cancers was detected), and the rate of missed diagnosis was up to 31% (1083 interval cancers occurred within 1 year of a negative endoscopy screening result). In addition, the positive predictive value of endoscopy screening was only 6.2%. In another study conducted in Japan, the sensitivity of prevalence screening was 0.955 (95% CI 0.875–0.991) for endoscopic screening and 0.893 (95% CI 0.718–0.977) for radiographic screening [16, 17].

In Japan and Korea, barium swallow continues to be one of the main choices for mass screening of gastric cancer; approximately 4,000,000 individuals undergo barium swallow testing annually In Japan and approximately 1,000,000 individuals undergo annual barium studies in Korea [1–4, 18, 19]. A serious drawback is low uptake of the target population; fear for radiation exposure (0.6 mSv), swallowing problems with the use of unpleasant barium meal, accidental fall during the examination and constipation (6%) causing rare but more serious complications such as intestinal obstruction or diverticulitis after examination may account for the reasons for this low uptake [1]. Furthermore, the performance of radiographic screening seems to be relatively poor and inefficient. In average, about 10% of the screened subjects are asked to take confirmatory endoscopic examinations, but gastric cancers are found in only about 1.5% of them [1]. Choi et al. [7] analyzed data on 1,765,909 subjects in Korea who underwent upper-gastrointestinal series mass-screening for gastric cancer. The sensitivity of upper-gastrointestinal series screening to detect gastric cancer was only 36.7% (1196 gastric cancers was detected), and the rate of missed diagnosis was up to 63.6% (2067 interval cancers occurred within 1 year of a negative upper-gastrointestinal series screening result). Although the detection rate of barium swallow testing for gastric polyps is high, the detection rate for lesions present as thickening of the gastric wall is low. Moreover, the majority of gastric polyps is benign and thought to be of no malignant potential; gastric cancers and precancerous gastric lesions (gastric mucosa with intestinal metaplasia or dysplasia) usually present as thickening of the gastric wall [20–24].

Etiology of gastric cancer includes Helicobacter pylori infection, diet and lifestyle, tobacco, alcohol and genetic susceptibility. Although a number of trials (especially in China and Taiwan) have demonstrated the possibility of cancer primary prevention through Helicobacter pylori screening and eradication [25, 26], it may not be cost-effective in areas of low risk [25]. More than 50% of the world population is infected with this bacterium; only less than 2% develop gastric cancer. Furthermore, Helicobacter pylori infection is more frequent in some countries such as India, Pakistan, and Bangladesh as compared to other Asian countries such as Japan, China and South Korea [26]. However, the frequency of gastric cancer is comparatively lower in India, Pakistan, and Bangladesh with that of Japan, China and South Korea. Such phenomenon of clinical diversity, defined as enigma, cannot be attributed to infection by Helicobacter pylori only [26, 27]. Other factors such as diet, tobacco and differences in the host genetic background in various ethnic groups may also play a role in its occurrence [26, 27]. Furthermore, the rate of eradication failure has dramatically risen in many countries due to resistance to antibiotic. Helicobacter pylori therapy in clinical practice is becoming progressively more difficult [28, 29]. Moreover, Helicobacter pylori antibody and serum pepsinogen tests are difficult to predict individuals who will not have gastric cancer in the future because of low predictive specificity of these tests [30]. Gastric cancer can still occur despite eradication and endoscopic follow-up might still be needed [31, 32].

In light of the remarkable advances in ultrasound technology, transabdominal ultrasound after oral administration of an echoic, cellulose-based, gastric ultrasound contrast agent (TUS-OCCA) has recently been suggested as a valuable initial screening tool for gastric diseases for the people who are not willing to undergo gastroscopy [8–15]. Zheng et al. [8] enrolled 383,945 patients with suspect gastric lesions who underwent complete oral contrast-enhanced gastric ultrasonography and endoscopic evaluation, the diagnostic performance of transabdominal ultrasound is not worse than upper-gastrointestinal endoscopy and can be used as a useful supplement to upper-gastrointestinal endoscopy.

The most important reason that TUS-OCCA is currently performed and continues to be investigated is that the pathological regression of various gastric lesions has become better understood during the recent 5 years, as follows: (1) the majority of gastric polyps is benign and thought to be of no malignant potential; and [20, 21] (2) precancerous gastric lesions are usually gastric mucosa with intestinal metaplasia or dysplasia, which presents as hypoechoic mucosal thickening [22–24]. The detection rate of TUS-OCCA for gastric polyps, which usually need no further mandatory treatment, is very low (approximately 20%), and the detection rate for hypoechoic thickening of the gastric wall, which does require further treatment, is high. Some comparative studies have reported that TUS-OCCA in the detection of gastric lesions (except for gastric polyps), with a sensitivity ranging from 76 to 100% and a specificity ranging from 94 to 100% [8–15]. The discrepancy between these studies may be due to the variable presence of overweight patients in the case series, the patients’ constitutions, the skill of the sonologists, and the features of gastric lesions (site, size, and ultrasonographic features). Generally, it is easier to reach a diagnosis when the tumor is located on the gastric antrum and gastric body, which is more accessible for examination than on the gastric fundus.

It should be noted that Su-RADS assessment categories was based on the thickening of gastric wall (especially the mucosal layer), it unavoidably miss some early gastric cancers without presenting as thickening of gastric wall. Although gastroscopy examination could detect some early gastric cancers without presenting as thickening of gastric wall (may present with mucosa changes), gastroscopy examination might miss some advanced gastric cancers without distinct mucosa damages (e.g., linitis plastica). The rates of gastric cancer missed by gastroscopy examination within 1 year have been reported up to be 31% [7]. The morphology of gastric cancers could be classified as polypoid, ulcerative, superficial spreading and diffuse infiltrative type (linitis plastica). For the gastric cancers with distinct mucosa damages, the patients may present with significant warning symptoms and will undergo gastroscopy examinations, and this cancer can fortunately be easily detected by gastroscopy examination. Moreover, for the gastric cancers without distinct mucosa damages, the patients may not present with significant warning symptoms and will not undergo gastroscopy examinations consciously, and these gastric cancers may not easily detected by gastroscopy examination. Those gastric cancers without distinct mucosa may present as gastric wall thickening, and can fortunately be easily detected by TUS-OCCA during population-based mass screening. Katai et al. [33] showed that the 5-year overall survival rates of gastric cancers with pathological stage IA, IB, II, IIIA, IIIB, and IV disease were 91.5, 83.6, 70.6, 53.6, 34.8, and 16.4%, respectively. The prognosis of gastric cancer is better if the gastric cancer could be detected earlier during population-based mass screening per year.

Because the percentage of patients with gastric cancers enrolled in this study was apparently higher than mass-screening population, the total patients’ malignant ratio (17.1%, 469/2738) enrolled in this study was apparently higher than mass-screening population. The percentage of patients with chronic gastritis in mass-screening population would be apparently higher than the patients enrolled in this study. Moreover, this unavoidable deviation does not significantly affect the sensitivity and specificity of Su-RADS categories for gastric cancer screening. As shown in Table 2, the malignant ratio in Su-RADS Categories 2 was 1.7% and the malignant ratio in Su-RADS Categories 3 was 12.2%. The differences of malignant ratios between Categories 2 and Categories 3 could indicate the different management; moreover, the indeed malignant ratio in Su-RADS Categories 2 and Categories 3 for mass-screening population would both be apparently lower. Therefore, for Su-RADS Categories 2, additional gastroscopy might be unnecessary; for Su-RADS Categories 3, additional gastroscopy may be necessary. Since TUS-OCCA cannot accurately distinguish between moderate–severe gastritis and gastric high-grade intraepithelial neoplasia (including early gastric cancer) in Su-RADS categories 3, additional gastroscopy recommendation may be appropriate. Although Su-RADS categories 3 to be considered as suspicion for malignant would decrease the positive predictive value of TUS-OCCA, performing additional gastroscopy for detecting gastritis in Su-RADS categories 3 are also appropriate. Compared with barium studies and gastroscopy screening, the estimated positive predictive values of barium studies and gastroscopy were 1.7 and 6.2%, respectively [7]. For Su-RADS categories 4 and categories 5 which malignant ratios were relatively high, endoscopy with biopsy should be recommended for them.

Although the anatomy of stomach varies greatly, the whole stomach could be divided into three parts (antrum, body and fundus-cardia) by the 5-step scanning procedure; nine serial standardized sections obtained by 5 steps could scan these three parts in transverse, longitudinal and coronal sections, respectively. For ultrasound doctors who have mastered the skills of abdominal ultrasound scanning, the learning time to master the skills of stomach ultrasound scanning is about 1–2 weeks. To simplify the ultrasound image interpretation, we first propose the Su-RADS system. The diagnostic criteria of Su-RADS were mainly based on thickness, which are objective and easily comprehend. Therefore, TUS-OCCA screening technique with Su-RADs system could be reproducible by other ultrasound doctors.

Besides barium studies, TUS-OCCA technique might be one of mass-screening tool for gastric cancers, especially for the individuals who are not willing to undergo gastroscopy examination. The Su-RADS system can inform the physicians about key findings, indicating the risk for malignancy and the necessity of additional gastroscopy examination. Prospectively randomly controlled study design with larger clinical trial is needed for further investigations.

## Electronic supplementary material

Below is the link to the electronic supplementary material.

Electronic Supplementary Material-Figure S1 Category 4A: Suspicious malignant finding. (a)(b) TUS-OCCA examination showing hypoechoic mucosa thickening (Gastric mucosa thickness was about 2.5-5 mm) (arrow). (c)(d) Gastroscopy examination showing congestion and eminence of gastric mucosa. Pathological diagnoses reveal early gastric cancer. (TIFF 4177 kb)

Electronic Supplementary Material-Figure S2 Category 4B: Suspicious malignant finding. (a) Gastric wall thickness was 9 mm (thick arrow), with the presence of 7 mm mucosa ulceration (thin arrow). (b) Gastroscopy examination showing congestion of gastric mucosa, with the presence of a 7 mm ulcerative lesion (thin arrow). Pathological diagnosis revealed a benign gastric ulcer. (TIFF 2379 kb)

Electronic Supplementary Material-Figure S3 Category 5B: Highly suggestive of malignant finding. (a) Gastric wall thickness was more than 10 mm (25–32 mm), with the presence of mucosal ulceration (arrow). (b) Gastroscopy examination showing a large gastric ulcerative lesion (about 8 cm) with bleeding. Pathological diagnosis revealed a gastric cancer. Unexpectedly, this patient had just underwent gastroscopy examination about 1.5 months ago, this lesion was missed. The reason why this lesion was missed by gastroscopy examination may be that this gastric cancer 1.5 months ago was a infiltrative-type cancer without distinct mucosa changes. The infiltrative-type gastric cancer changed into a ulcerative-type cancer with the tumor tissue necrosis. (TIFF 2992 kb)

## Abbreviations

Su-RADS: The stomach ultrasound reporting and data system
TUS-OCCA: Transabdominal ultrasound after oral administration of an echoic, cellulose-based, gastric ultrasound contrast agent
