# Supplementary figures and images for: Preliminary opinion on assessment categories of stomach ultrasound report and data system (Su-RADS)
Source: Gastric Cancer. 2018 Jan 25;21(5):879–88. doi: 10.1007/s10120-018-0798-x (PMC6097085; doi:10.1007/s10120-018-0798-x)

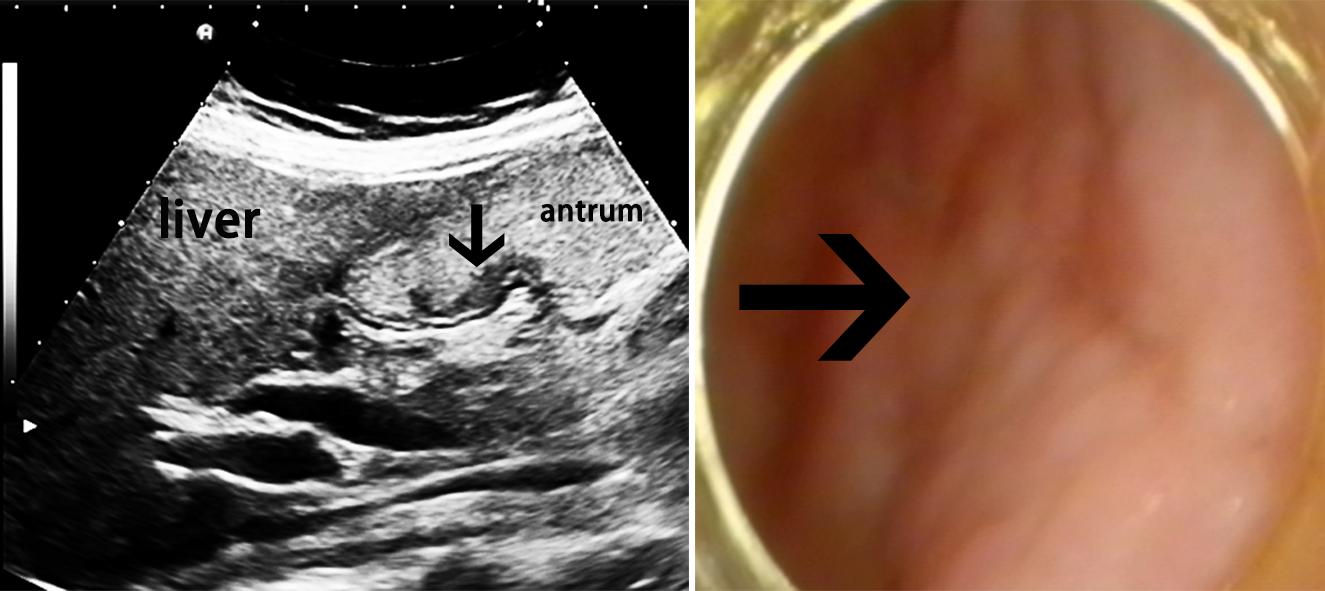

Supplement: Supplementary file 1 — Electronic Supplementary Material-Figure S1 Category 4A: Suspicious malignant finding. (a)(b) TUS-OCCA examination showing hypoechoic mucosa thickening (Gastric mucosa thickness was about 2.5-5 mm) (arrow). (c)(d) Gastroscopy examination showing congestion and eminence of gastric mucosa. Pathological diagnoses reveal early gastric cancer. (TIFF 4177 kb) [file 10120_2018_798_MOESM1_ESM.tif]

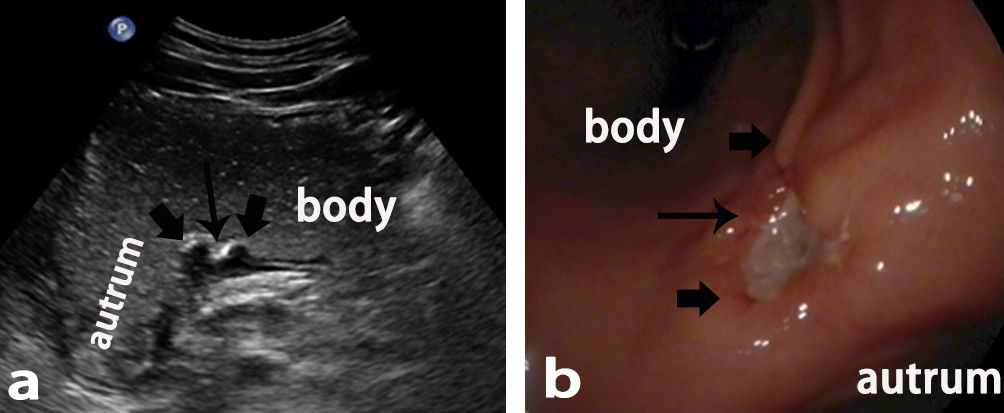

Supplement: Supplementary file 2 — Electronic Supplementary Material-Figure S2 Category 4B: Suspicious malignant finding. (a) Gastric wall thickness was 9 mm (thick arrow), with the presence of 7 mm mucosa ulceration (thin arrow). (b) Gastroscopy examination showing congestion of gastric mucosa, with the presence of a 7 mm ulcerative lesion (thin arrow). Pathological diagnosis revealed a benign gastric ulcer. (TIFF 2379 kb) [file 10120_2018_798_MOESM2_ESM.tif]

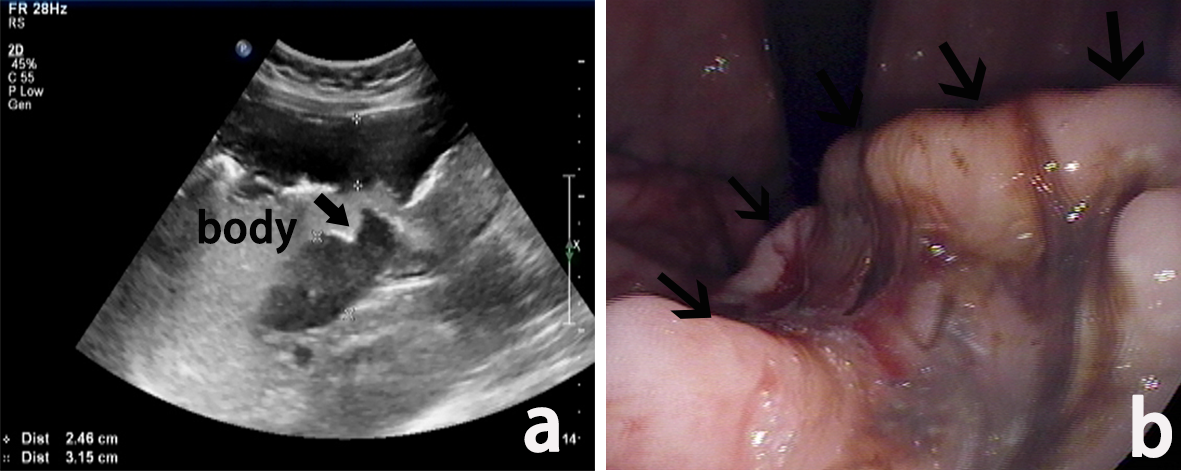

Supplement: Supplementary file 3 — Electronic Supplementary Material-Figure S3 Category 5B: Highly suggestive of malignant finding. (a) Gastric wall thickness was more than 10 mm (25–32 mm), with the presence of mucosal ulceration (arrow). (b) Gastroscopy examination showing a large gastric ulcerative lesion (about 8 cm) with bleeding. Pathological diagnosis revealed a gastric cancer. Unexpectedly, this patient had just underwent gastroscopy examination about 1.5 months ago, this lesion was missed. The reason why this lesion was missed by gastroscopy examination may be that this gastric cancer 1.5 months ago was a infiltrative-type cancer without distinct mucosa changes. The infiltrative-type gastric cancer changed into a ulcerative-type cancer with the tumor tissue necrosis. (TIFF 2992 kb) [file 10120_2018_798_MOESM3_ESM.tif]
